# Skin Responses in Newly Diagnosed Polyneuropathy, Organomegaly, Endocrinopathy, Monoclonal Gammopathy, and Skin Changes (POEMS) Syndrome After Therapy With Low-Dose Lenalidomide Plus Dexamethasone

**DOI:** 10.3389/fimmu.2021.681360

**Published:** 2021-05-06

**Authors:** Yajuan Gao, Shiyu Zhang, Lu Yang, Jian Li, Yuehua Liu, Tao Wang

**Affiliations:** ^1^ Department of Dermatology, Peking Union Medical College Hospital, Chinese Academy of Medical Sciences and Peking Union Medical College, Beijing, China; ^2^ Department of Hematology, Peking Union Medical College Hospital, Chinese Academy of Medical Sciences and Peking Union Medical College, Beijing, China

**Keywords:** POEMS syndrome, skin changes, vascular endothelial growth factor, lenalidomide, dexamethasone, prospective studies

## Abstract

**Background:**

Polyneuropathy, organomegaly, endocrinopathy, monoclonal gammopathy, and skin changes (POEMS) syndrome is a rare paraneoplastic disease with a high prevalence of skin symptoms. Few studies have focused on skin responses to systemic treatment of this disease.

**Objective:**

To evaluate skin responses after treatment with low-dose lenalidomide plus dexamethasone and determine their relationship with vascular endothelial growth factor (VEGF) and hematological responses.

**Methods:**

A total of 41 consecutive Chinese patients who were at least 18 years of age with newly diagnosed POEMS syndrome were enrolled. 36 of them completed 12 cycles of treatment. Skin, serum VEGF, hematological and neurological responses were documented at 1, 2, 3, 6, 9, and 12 months during treatment.

**Results:**

Skin manifestations were not associated with serum VEGF levels at baseline. Of the patients with hyperpigmentation, hemangiomas, hypertrichosis, white nails, or acrocyanosis separately, 26/34 (76.5%), 11/30 (36.7%), 14/15 (93.3%), 21/21 (100%), and 4/4 (100%) achieved complete responses at 12 months. Reduction in hyperpigmentation after 12 months reflected a good VEGF response (*P* = 0.017).

**Conclusion:**

Low-dose lenalidomide plus dexamethasone therapy is effective in reversing skin changes in patients with POEMS syndrome.

**Clinical Trial Registration:**

Clinicaltrials.gov, identifier NCT01816620

## Introduction

Polyneuropathy, organomegaly, endocrinopathy, monoclonal gammopathy, and skin changes (POEMS) syndrome is a rare multisystem paraneoplastic disease with plasma cell dyscrasia (1). Skin changes are a minor component of the diagnostic criteria, and large retrospective studies in America, Japan, China, and England have indicated that skin changes can be present in 68, 84, 89, and 69% of POEMS patients, respectively (2–5). Cutaneous manifestations mainly include hyperpigmentation, glomeruloid hemangiomas, hypertrichosis, rubor and acrocyanosis, white nails, sclerodermoid changes, facial atrophy, flushing, or clubbing fingers (4, 6–8).

The pathogenesis of POEMS syndrome is still unclear, but vascular endothelial growth factor (VEGF) is considered to be an important factor related with disease activity (9) and prognosis (10). However, only one study (6) has examined the relationship between VEGF levels and skin manifestations at baseline and after therapy. In that study, the median VEGF levels only showed difference between patients with and without hypertrichosis (*P* = 0.04). After autologous peripheral blood stem cell transplantation (aPBSCT) in nine patients, a significant relationship was only observed between VEGF level decreases and response of hypertrichosis (*P* = 0.007). Some clinical studies (11, 12) and cases (9, 13–15) reported skin responses with overall improvement after systemic therapies but did not assess possible correlations of skin responses with VEGF levels. Therefore, we still know little about skin responses after systemic treatments of POEMS syndrome.

Lenalidomide, a thalidomide analog, has shown its anti-VEGF and immunoregulation effects in treatment of POEMS syndrome. Here, we report the results of a prospective study designed to investigate skin responses in patients with newly diagnosed POEMS syndrome after therapy with low-dose lenalidomide plus dexamethasone.

## Methods

### Patients

This was a single-center, open-label, single-arm, prospective pilot study approved by the ethics committee of Peking Union Medical College Hospital and conducted in accordance with the Declaration of Helsinki. Written informed consents were obtained from all patients. A total of 41 consecutive patients who were newly diagnosed with POEMS syndrome between April 2014 and November 2015 were enrolled. All patients met the diagnostic criteria for POEMS syndrome defined by Dispenzieri (16), namely, two mandatory criteria (polyneuropathy and monoclonal plasma cell-proliferative disorder), at least one major criterion (sclerotic bone lesions, Castleman disease, or elevated serum VEGF concentration), and one minor criterion (organomegaly, extravascular volume overload, endocrinopathy, skin changes, papilledema, or thrombocytosis/polycythemia). For the AESOP syndrome (adenopathy and extensive skin patch overlying a plasmacytoma) (17), considering the efficacy of removal of plasmacytoma, surgery or radiotherapy may be a better choice than systemic treatment in these patients. Therefore, the AESOP patients were ruled out of this study.

### Treatment

Patients were administered 12 cycles of oral lenalidomide (Revlimid; Celgene, Summit, NJ, USA) at 10 mg/day for 21 days of the 28-day cycle and oral dexamethasone at 40 mg/week. Aspirin at 100 mg/day was prescribed to prevent thrombotic events.

### Evaluation of Skin Symptoms

Before treatment, all patients underwent a thorough skin examination by two of the authors (YL and TW) who are experienced dermatologists. Skin changes were examined every month for the first 3 months of treatment and every 3 months thereafter until the end of therapy. Photographs were taken when necessary. A complete response (CR_S_) of a skin manifestation (except hemangiomas) was defined as the complete disappearance of the lesion and return of the skin to a state before disease onset, as determined by the patient’s dermatologist, the patient’s recall of events, or by comparison with photographs taken before the skin changes first emerged. Given that pigmentation left by hemangiomas usually requires quite a long period to fade, CR_S_ for hemangiomas were defined as flattening of all hemangiomas regardless of pigmentation. Considering that with or without hemangioma is the main evaluation indicator of treatment efficacy in this study, we did not assess the morphological features of all hemangiomas in one patient, which is also hard to realize in the clinical at the most time. Acquired facial lipoatrophy and skin thickening were not assessed in this study because of the difficulty in obtaining precise measurements at each examination and the unreliability of patient-reported self-assessments. The time to response was defined as the date of treatment initiation to the date at which complete reversal of one skin symptom was observed.

For patients with hemangiomas, skin biopsies were taken at baseline and sectioned for anti-VEGF immunohistochemical staining. Primary and secondary antibodies were purchased from Santa Cruz Biotechnology (Santa Cruz, CA, USA), and color development was achieved using diaminobenzidine solution. Sections were counterstained with hematoxylin, dehydrated, mounted, and visualized. Negative control sections were prepared by substitution of the primary antibody with a control IgG. Hemangiomas from three of 11 patients who were still with them at the end of therapy were also biopsied and immunostained for VEGF.

### Laboratory and Clinical Assessments

Blood samples were collected at baseline and 1, 2, 3, 6, 9, and 12 months. Serum VEGF concentrations were determined using a human Quantikine ELISA Kit (R&D Systems, Minneapolis, MN, USA). A complete response for VEGF (CR_V_) is defined as normalization of the VEGF level (<600 pg/ml); partial response (PR_V_) was defined as ≥50% reduction in serum VEGF but the concentration remained >600 pg/ml. Stable disease (SD_V_) was defined as VEGF levels not meeting the criteria for CR_V_ or PR_V_. Hematological responses were assessed by serum protein electrophoresis and serum and urine immunofixation electrophoresis. Two hematological responses were defined: no response (N_H_) and complete response (CR_H_), which is negative immunofixation of the serum and urine.

The overall neuropathy limitation scale (ONLS) was used to assess neurologic disability (18). The ONLS ranges from 0 (no disability) to 12 (maximal disability) and consists of an arm score and a leg score. The arm score ranges from 0 (normal) to 5 (disability in both arms preventing all purposeful movement) and the leg score ranges from 0 (normal) to 7 (restricted to wheelchair or bed most of the day, unable to make any purposeful movements of the legs). A neurologic response was defined as a reduction in ONLS score of ≥1.

Other assessments included organomegaly (based on computed tomography); extravascular volume overload (ascites and pleural effusions based on computed tomography; peripheral edema based on clinical examination), systolic pulmonary artery pressure (sPAP, echocardiography), renal function (estimated glomerular filtration rate, eGFR) and thyroid function (FT4, TSH, and TT4 levels).

### Statistical Analysis

Data was analyzed with SPSS 26.0 software (SPSS, Chicago, IL, USA). Student’s t test or the Mann–Whitney U test was used to analyze group differences for continuous variables, and Pearson’s χ2 test or Fisher’s exact test were used to analyze group differences for categorical variables. A two-tailed *P* value of <0.05 was considered significant for all analyses.

## Results

### Baseline Characteristics

**Figure 1:**
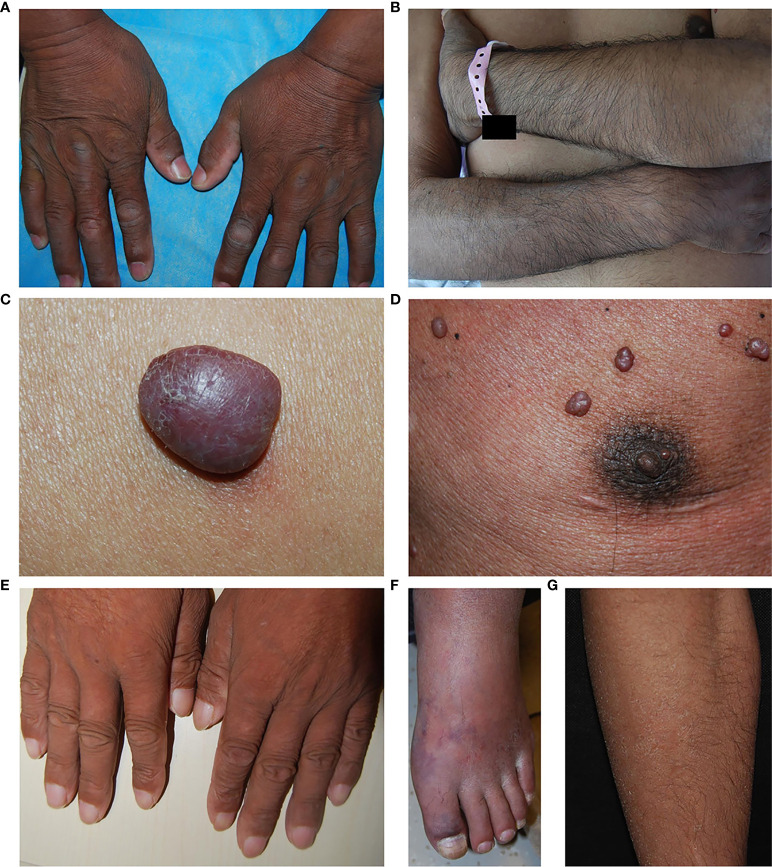
Skin changes in patients with POEMS syndrome. **(A)** Hyperpigmentation. **(B)** Hypertrichosis. **(C)** A typical hemangioma. **(D)** Hemangiomas with darkened mammary areola. **(E)** White nails. **(F)** Acrocyanosis. **(G)** Dry skin and ichthyosis-like lesions.

A total of 41 patients (28 men, 13 women) were enrolled in this study. The baseline clinical features are shown in **Table 1**. The median age at diagnosis was 49 (range, 21–70) years. Three patients died from disease-related causes, and two patients withdrew during the study. At baseline, only one patient had normal serum VEGF concentration and the median VEGF was 5,155 (range 534–14,328) pg/ml. All 41 patients had skin manifestations. Hyperpigmentation was observed in 38 patients (92.7%); of these, 25 patients (65.8%) were affected over the whole body and 13 (34.2%) on sun-exposed areas. Hemangiomas were found in 33 patients (80.5%), with locations on the trunk (all patients), extremities (three patients, 9.1%), scalp (two patients, 6.1%), and face (one patient, 3.0%). 25 patients (61%) had white nails and all of them were Terry’s nails (19), with whitening of all finger nails and disappearance of lunula, leaving a narrow pink band at the tips; hypertrichosis was in 15 patients (36.6%), most prominently on the pretibial region of the lower legs; and acrocyanosis was present in 6 patients (14.6%). Nine patients (22.0%) exhibited dry skin and ichthyosis-like lesions, especially on lower legs. Representative photographs of some typical skin symptoms are shown in **Figure 1**. Median time from onset of skin changes to diagnosis is 18 months (3–84 months), 18 months (4–60 months) and 12 months (3–24 months) for hyperpigmentation, hemangiomas, and hypertrichosis separately.

**Table 1:** Baseline characteristics of 41 patients with newly diagnosed POEMS syndrome.

Baseline	N (%)	Median (range)
Male	28 (68)	NA
Age at diagnosis, years	NA	49 (21–70)
Time from symptoms to diagnose, months	NA	7 (1-84)
Polyneuropathy	41 (100)	NA
ONLS score		
Upper limb	NA	1 (0–4)
Lower limb	NA	2 (1–7)
Overall	NA	4 (1–10)
Organomegaly	41 (100)	NA
Lymphadenopathy	38 (93)	NA
Splenomegaly	22 (54)	NA
Hepatomegaly	12 (29)	NA
Endocrinopathy	41 (100.0)	NA
Diabetes mellitus	6 (15)	NA
Hypothyroidism	17 (42)	NA
Gynecomastia (Male)	21/28 (75)	NA
Increased ACTH	28/39 (72)	NA
M-protein	41 (100.0)	NA
IgA*λ*	25 (61)	NA
IgA*λ* + IgG*κ*	1 (2)	NA
IgA*λ* + IgM*λ*	1 (2)	NA
IgGλ	13 (32)	NA
* λ*	1(2)	NA
Skin change	41 (100.0)	NA
Hyperpigmentation	38 (93)	NA
Hemangiomas	33 (81)	NA
White nails	25 (61)	NA
Hypertrichosis	15 (37)	NA
Acrocyanosis	6 (15)	NA
Dry skin and ichthyosis-like lesions	9 (22)	NA
Bone lesion	31 (76)	NA
Extravascular overload	40 (98)	NA
Edema	39 (95)	NA
Ascites	17 (42)	NA
Pleural effusion	21 (51)	NA
Periscardial effusion	28 (68)	NA
Serum VEGF, pg/ml	40 (98)	5155 (534–14328)
Pulmonary hypertension, %	3 (7)	54 (51–93)
Papilledema	23 (56)	NA
Erythrocytosis	8 (20)	138 (94–182)
Thrombocytosis	18 (44)	332 (89–628)

NA, not available; ONLS, overall neuropathy limitation scale; ACTH, adrenocorticotropic hormone; VEGF, vascular endothelial growth factor.

### Relationships Between Skin Manifestations, Serum VEGF, and Other Symptoms at Baseline

**Figure 2:**
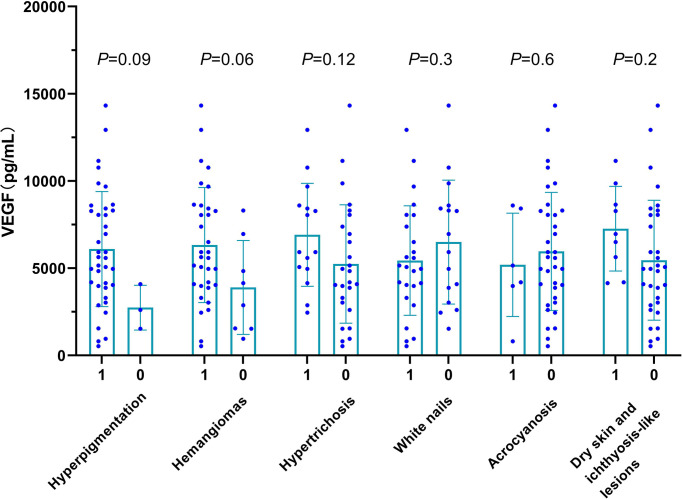
Baseline serum VEGF levels in patients with (1) or without (0) specific skin changes. Skin changes include hyperpigmentation (*P* = 0.09), hemangiomas (*P* = 0.06), hypertrichosis (*P* = 0.12), white nails (*P* = 0.32), acrocyanosis (*P* = 0.60), dry skin and ichthyosis-like lesions (*P* = 0.15).

To determine whether skin manifestations and serum VEGF levels correlate at baseline, we stratified the patients according to the presence or absence of specific skin manifestations. As shown in **Figure 2** and **Supplementary Table 1**, serum VEGF levels were not significantly associated with any of the cutaneous symptoms. However, trends approaching significance were detected between higher VEGF levels and the presence of hyperpigmentation (median 6,099 pg/ml *vs.* 2,741 pg/ml, *P* = 0.09), hemangiomas (6,921 pg/ml *vs.* 5,243 pg/m*L*, *P* = 0.06), and hypertrichosis (6,328 pg/ml *vs*. 3,896 pg/ml, *P* = 0.06). Adrenal insufficiency is common in POEMS syndrome (1) and may cause hyperpigmentation. However, comparison of serum adrenocorticotropic hormone (ACTH) levels (**Supplementary Table 2**) showed no significant differences between patients with or without hyperpigmentation (*P* = 0.53). Therefore, hyperpigmentation in our patients was more likely to be a result of elevated levels of VEGF and/or other cytokines rather than adrenal insufficiency.

We also investigated potential relationships between skin and systemic manifestations at baseline. Although the differences were not clinically meaningful, platelet levels were elevated in patients with hemangiomas (353.0 × 10^9^/ml *vs*. 248.5 × 10^9^/ml, *P* = 0.008) and decreased in patient with white nails (293.0 × 10^9^/ml *vs*. 362.5 × 10^9^/ml, *P* = 0.023) (**Supplementary Table 3**). The presence of Terry’s nails was significantly associated with restrictive ventilatory dysfunction (*P* = 0.020), subclinical and clinical hypothyroidism (*P* = 0.022), and ascites (*P* = 0.013) (**Supplementary Table 4**). No significant relationships were observed between skin symptoms and ONLS score, M protein type, organomegaly, endocrinopathy, extravascular fluid overload, lung function, sPAP, renal function, papilledema, hemoglobin, or interleukin (IL)-6 level at diagnosis.

### Skin Responses After 12 Months of Therapy

**Figure 3:**
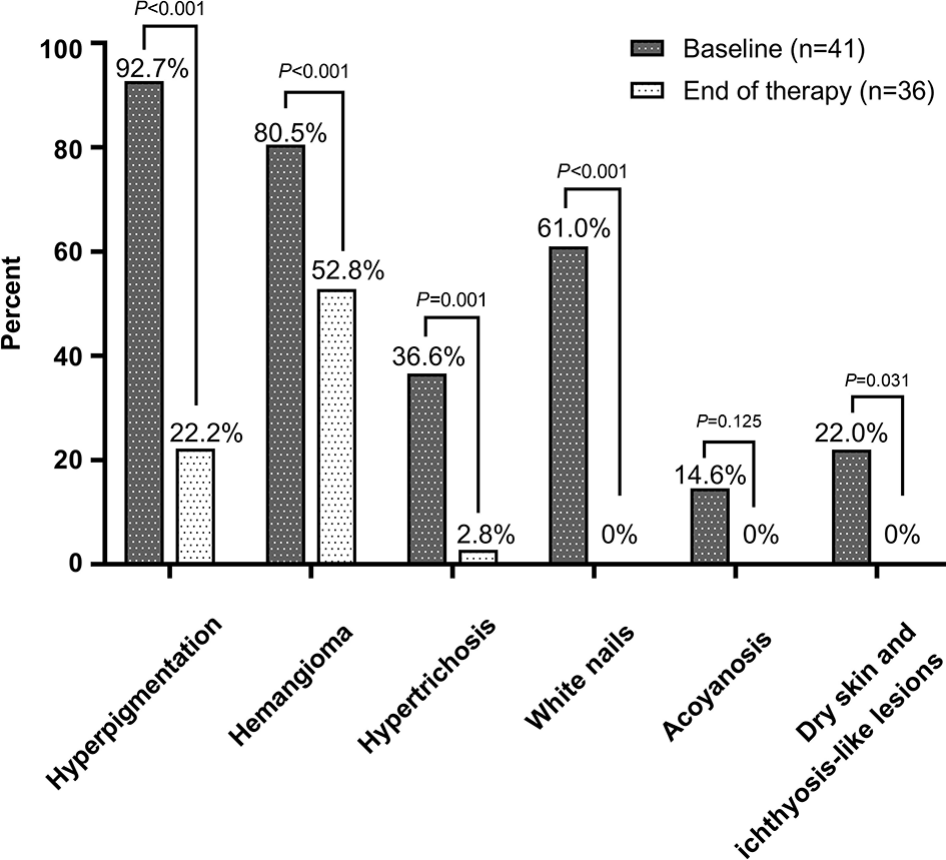
Percentage of different skin changes in POEMS patients at the baseline (n = 41) and the end of therapy (n = 36).

At the end of the 12 months, the number and proportion of patients who achieved CR_S_ for hyperpigmentation, hemangiomas, hypertrichosis, white nails, and acrocyanosis was 26/34 (76.5%), 11/30 (36.7%), 14/15 (93.3%), 21/21 (100%), and 4/4 (100%), respectively (**Figure 3**). The time to 50% remission was the shortest (1.5 months) for acrocyanosis, followed by white nails (2 months), hyperpigmentation, and hypertrichosis (both 6 months), and glomerular hemangioma (>12 months). Typical pictures of hyperpigmentation, hemangiomas and hypertrichosis in patients achieved CRs are shown in **Supplementary Figure 1**.

To determine the significance of the skin responses, we examined the associations between hyperpigmentation, hemangioma, and hypertrichosis responses and VEGF, hematological and neurological responses **(** **Table 2** **)**. Notably, the hyperpigmentation response is significantly associated with the VEGF response (*P* = 0.017), whereas no other associations were detected. White nails and acrocyanosis were not included in this analysis because the response rates were 100%. The hyperpigmentation response was the only skin response to significantly involve improvement of thyroid function (*P* = 0.025, **Supplementary Table 5**). Detailed information about the therapeutic reaction in every patient can be found in **Supplementary Table 6** and our previously published articles (20, 21).

**Table 2:** Skin responses and their relationships with VEGF, hematological and neurological responses at the end of therapy.

Skin responses	VEGF responses, number of patients (%)				Hematological responses, number of patients (%)			Neurological responses, number of patients (%)		
	CR_V_	PR_V_	SD_V_	*P*	CR_H_	N_H_	*P*	R_N_	N_N_	*P*
Hyperpigmentation (n=34)										
Response	15 (44.1)	10 (29.4)	1 (2.9)	0.017	12 (35.3)	14 (41.2)	1.000	24 (70.6)	0	–
No response	1 (2.9)	4 (11.8)	3 (8.8)		4 (11.8)	4 (11.8)		8 (23.5)	0	
Hemangiomas (n=30)										
Response	8 (26.7)	8 (26.7)	3 (10)	0.568	6 (20)	5 (16.7)	1.000	10 (33.3)	0	1.000
No response	6 (20)	5 (16.7)	0 (0)		10 (33.3)	9 (30)		17 (56.7)	1 (3.3)	
Hypertrichosis (n=15)										
Response	7 (46.7)	7 (46.7)	0 (0)	1.000	7 (46.7)	7 (46.7)	1.000	13 (86.7)	0	–
No response	0 (0)	1 (6.7)	0 (0)		1 (6.7)	0 (0)		1 (6.7)	0	
All patients (n=36)										
	17 (47.2)	14 (38.9)	4 (11.1)^a^	–	17 (47.2)	19 (52.8)	–	33 (91.7)^b^	1 (2.8)	–

VEGF, vascular endothelial growth factor; CR_V_, complete VEGF response (VEGF level returns to normal [<600 pg/ml]); PR_V_, partial VEGF response (VEGF decreased ≥50% but remains above normal); SD_V_, stable disease (does not meet criteria for CR_V_ or PR_V_); CR_H_, complete hematologic response (negative immunofixation of serum or urine); N_H_, no hematologic response (does not meet CR_H_ criteria); R_N_, neurological response (a reduction in ONLS score of ≥1); N_N_, no neurological response (does not meet R_N_ criteria). ^a^VEGF level of one patient was normal at the baseline, so this patient’s VEGF response is not included here. ^b^2 patients with 0 ONLS score at the baseline are not included here.

### Biopsy of Hemangiomas Before and After Treatment

**Figure 4:**
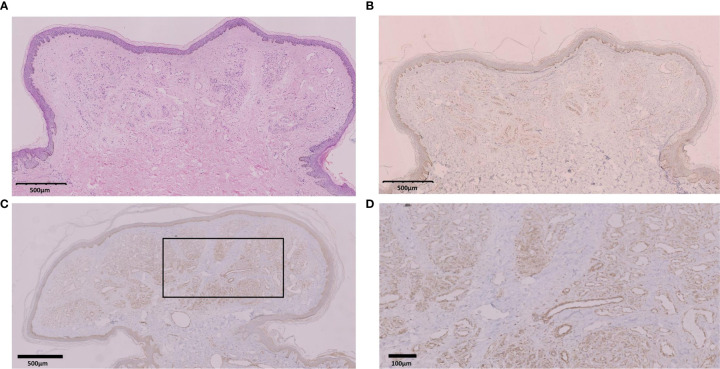
**(A)** Hematoxylin and eosin (H&E) of a polypoid neoplasm taken from a POEMS patient with hemangiomas at the baseline. Scale bars, 500 μm. **(B)** VEGF immunostaining of the same hemangioma in **(A)** Scale bars, 500 μm. **(C)** VEGF immunostaining of a hemangioma taken at the end of therapy. **(D)** Magnification of a section shown in **(C)**. VEGF staining is strongly positive in hyperplastic endothelial cells with epidermal atrophy and dilated capillary networks. Scale bars, 100 μm.

Biopsies were taken from all patients with hemangiomas (n = 33) at baseline as well as the three patients in whom hemangiomas were still present at the end of treatment. Histopathological examination revealed complex capillary networks, with dilated vessels and glomerulus-like hemangiomas in the dermis and hyperplasia of vascular endothelial cells in all samples (**Figure 4**). Immunostaining of biopsy sections revealed strong VEGF expression in vascular endothelial cells. VEGF expression in hemangiomas remaining at 12 months was comparable to that seen at baseline.

## Discussion

This is the first cohort study with the largest sample size to evaluate skin responses in newly-diagnosed POEMS syndrome after therapy of low-dose lenalidomide plus dexamethasone since now. Our results demonstrated that this combination strategy is effective in reversing skin manifestations of POEMS syndrome.

Cutaneous manifestations are some of the most notable features of POEMS syndrome. Because they can be assessed at the initial presentation without laboratory examinations, skin lesions can make a valuable contribution to the early diagnosis of this rare multisystemic disease. For patients who relapse or progress after first-line treatment, skin changes can also be a clue to disease activity (12). In our center, we reviewed 120 patients who experienced relapse after first-line therapy and found that 82 (68.3%) patients had recurrence of cutaneous symptoms (unpublished data). Thus, understanding how skin changes reflect the presentation and progress of this disease and its response to therapy may improve both the diagnosis and treatment of POEMS syndrome.

Elevated serum VEGF levels are known to contribute to characteristic symptoms of POEMS syndrome, including extravascular fluid overload, hemangiomas, and papilledema, by increasing the proliferation of endothelial cells and the permeability of the vessel wall. In our study, VEGF expression in skin sections was particularly strong in vascular endothelial cells, with hyperplasia of the complex capillary network, underscoring the important role of VEGF in skin changes. However, there were no significant correlations between skin symptoms and VEGF levels in our cohort, which was expected because VEGF levels do not usually reflect clinical symptoms. Patients with POEMS syndrome tend to have a higher skin vessel density than healthy individuals, but the density itself is not related to VEGF levels (22). The anti-VEGF antibody bevacizumab has been tested for the treatment of POEMS syndrome, but the results were ambiguous (23–32). Skin responses to bevacizumab therapy can be good or remain stable in different reports. Besides VEGF, other pro-angiogenic and pro-inflammatory factors, such as basic fibroblast growth factor, hepatocyte growth factor, tumor necrosis factor-α, IL-6, and IL-12, are elevated in POEMS syndrome (33–37). In a previous study, IL-6 expression was detected in endothelial cells in two skin samples from three patients (38). It highlights that other cytokines may also contribute to skin changes in POEMS syndrome. Deposition of IgG, IgA, IgM and *κ* and *λ* light chain globulins have been found in periodic acid–Schiff-positive eosinophilic globules within endothelial cells in hemangiomas (39), suggesting the probable participation of M protein in skin lesions.

Hyperpigmentation is usually the most prevalent skin characteristic of POEMS syndrome (2–4). In the present study, improvement in this symptom was the most closely related to the VEGF response and also correlated with improvement in thyroid function after therapy. Therefore, stabilization or progression of skin pigmentation during therapy may be indicative of a poor VEGF response and may also be a clue for relapsed or refractory disease. The appearance of hemangiomas, which usually occurs on the trunk, is a unique characteristic skin change in patients with POEMS syndrome. We noticed softening and flattening of hemangiomas during therapy, but these lesions were the slowest of all skin manifestations to show improvement or eradication. The hemangiomas remained at the end of the 12-month study showed strong VEGF staining, suggesting that a short period of systemic therapy may not be sufficient to completely deplete cytokines deposited in skin tissue. Laser resection is often helpful for patients who desire hemangioma removal for cosmetic reasons. Local application of bevacizumab is a potential strategy for skin lesions, because improvement of optic disc edema is detected upon local bevacizumab injection in some patients (40–42). This potential route of therapy needs to be investigated.

Few studies have focused on skin responses in patients with POEMS syndrome after systemic therapy. Barete et al. evaluated the skin responses in nine patients after aPBSCT (6) and found that a decrease in serum VEGF levels correlated significantly only with improvement in hypertrichosis. However, we cannot be sure whether this conclusion is related with therapy choices or due to small sample size. Of the nine patients, two achieved complete reversal of skin changes and seven remained stable; however, the response rates of hyperpigmentation, hypertrichosis, acrocyanosis, and white nails were relatively high at 83.3, 80, 75, and 100%, respectively. A patient with smoldering myeloma with clinical presentation of POEMS syndrome showed complete recover of skin changes at 1 year after initiation of lenalidomide and dexamethasone therapy (14). In our study, all skin changes except hemangiomas showed good responses by 12 months, suggesting that low-dose lenalidomide plus dexamethasone was effective in reversal of skin changes in patients with POEMS syndrome. In addition to aPBSCT and lenalidomide, other treatment options include local radiotherapy, melphalan, thalidomide, and bortezomib. Whether consistent skin responses are observed with different treatment strategies is a question for further study.

This study has some limitations. First, this is a single-center trial with a small number of patients, due largely to disease rarity. Second, no control arm was included because effectiveness of dexamethasone alone is rather limited in POEMS syndrome. Third, the observation period was short and there may be a lag before a response in skin symptoms is seen, especially hemangiomas. Finally, skin evaluations were open labeled to two dermatologists at the baseline and it may be better to keep skin examination results blind for them.

## Conclusion

Skin changes do not reflect serum VEGF levels at baseline in patients with POEMS; however, remission of hyperpigmentation during therapy is associated with a good VEGF response. Low-dose lenalidomide plus dexamethasone combination therapy is effective in reversing skin changes in patients with POEMS syndrome.

## Data Availability Statement

The datasets presented in this article are not readily available because data is participants identifiable. Requests to access the datasets should be directed to YG, gaoyj188@foxmail.com.

## Ethics Statement

The studies involving human participants were reviewed and approved by Peking Union Medical College Hospital. The patients/participants provided their written informed consents to participate in this study. Written informed consent was obtained from the individual(s) for the publication of any potentially identifiable images or data included in this article.

## Author Contributions

TW, JL, and YL designed the study and contributed to data acquisition. SZ and LY were involved in data acquisition. YG contributed to data analysis and drafting of the manuscript. TW and YL contributed to critical revision of the manuscript and supervision of the study. All authors contributed to the article and approved the submitted version.

## Funding

This work had been supported by the National Natural Science Foundation of China (no. 81570195, to JL), the Beijing Natural Science Foundation (no. 7142130, to JL), the Specialized Research Fund for the Doctoral Program of Higher Education (no. 2013110611000, to JL), CAMS Innovation Fund for Medical Sciences (CIFMS, no. 2016-I2M-1-002, to JL), the National Key Research and Development Program of China (no. 2016YFC0901503, to JL), the Fundamental Research Funds for the Central Universities (no.3332018025, to TW), NCMI-ABD02-201709 (to TW), Beijing Dongcheng District Excellent Talent Support Training project (no.2019JGM-5, to TW), the National Key Research and Development Program of China Grant (no.2016YFC0901500), and the Center for Rare Diseases Research, Chinese Academy of Medical Sciences, Beijing, China. There is no involvement of the funder in study design, data collection, data analysis, and manuscript preparation.

## Conflict of Interest

The authors declare that the research was conducted in the absence of any commercial or financial relationships that could be construed as a potential conflict of interest.
