# Supplementary material for: Skin Responses in Newly Diagnosed Polyneuropathy, Organomegaly, Endocrinopathy, Monoclonal Gammopathy, and Skin Changes (POEMS) Syndrome After Therapy With Low-Dose Lenalidomide Plus Dexamethasone
Source: Front Immunol. 2021 May 6;12:681360. doi: 10.3389/fimmu.2021.681360 (PMC8134691; doi:10.3389/fimmu.2021.681360)
Supplement: Supplementary file 1 [file DataSheet_1.docx]

Supplementary figure 1. Complete responses in hyperpigmentation, hemangioma and hypertrichosis. (A) and (B) shows hyperpigmentation before and after treatment, respectively.

(C) and (D) shows change of a hemangioma before and after treatment.

(E) and (F) shows reversal of hypertrichosis before and after treatment.


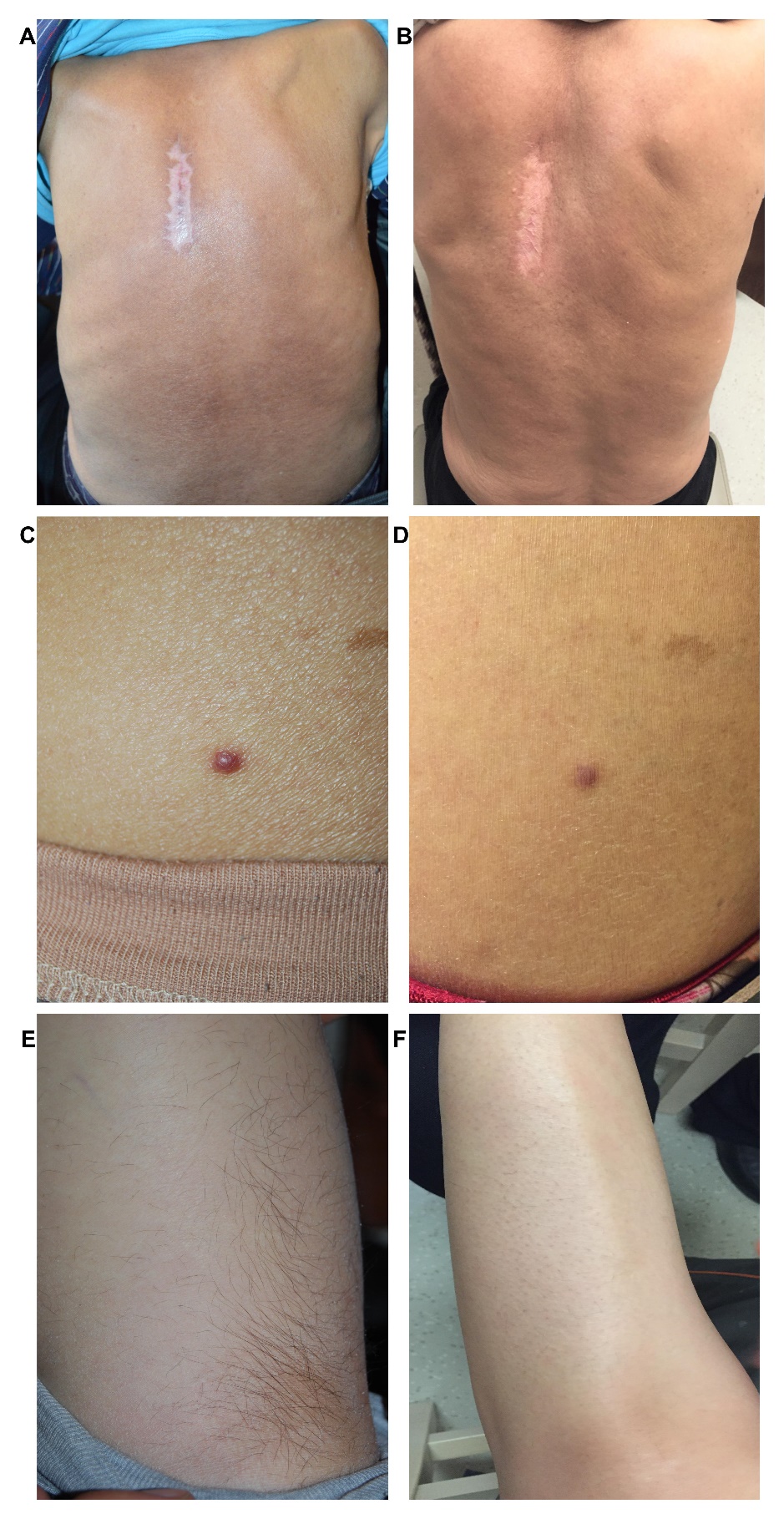


Supplementary table 1. Baseline VEGF levels in POEMS patients with or without specific skin changes.

| Skin changes | VEGF, pg/mL, median (range) | *P* |
| --- | --- | --- |
| Hyperpigmentation | | |
| Yes | 6099（534-14328） | 0.09 |
| No | 2741（1533-4086） |  |
| Hemangioma | | |
| Yes | 6328（534-14328） | 0.06 |
| No | 3896（955-8306） |  |
| Hypertrichosis | | |
| Yes | 6912（2459-12936） | 0.12 |
| No | 5243（534-14328） |  |
| White nails | | |
| Yes | 5439（534-12936） | 0.32 |
| No | 6501（1523-14328） |  |
| Acrocyanosis | | |
| Yes | 4676.5 (810-8594) | 0.60 |
| No | 5590 (534-14328) |  |
| Dry skin and ichthyosis-like lesions | | |
| Yes | 6962 (534-14328) | 0.15 |
| No | 4965 (4150-11154) |  |

*VEGF*, vascular endothelial growth factor; *POEMS* syndrome, polyneuropathy, organomegaly, endocrinopathy, monoclonal, gammopathy, and skin changes syndrome.

Supplementary table 2. Baseline adrenocorticotropic hormone (ACTH) levels in POEMS patients with or without hyperpigmentation.

| Skin changes | ACTH, pg/mL, median (range) | *P* |
| --- | --- | --- |
| Hyperpigmentation | | |
| Yes | 87.02（14.6-318.0） | 0.53 |
| No | 58.63（11.5-100.0） |  |

*ACTH*, adrenocorticotropic hormone.

Supplementary table 3. Baseline platelet (PLT) levels according to hemangioma and white nails in patients with POEMS syndrome.

| Skin changes | PLT, ×10^9^/L, median (range) | *P* |
| --- | --- | --- |
| Hemangioma | | |
| Yes | 353.0 (187-628) | 0.008 |
| No | 248.5 (89-361) |  |
| White nails | | |
| Yes | 293.0 (89-467) | 0.023 |
| No | 362.5 (206-628) |  |

*PLT*, platelet; *POEMS* syndrome, polyneuropathy, organomegaly, endocrinopathy, monoclonal, gammopathy, and skin changes syndrome.

Supplementary table 4. Skin changes of POEMS syndrome and closely related symptoms at the baseline.

| Skin changes | Associated symptoms | Presence of manifestations, number of patients (%) | | | | *P* |
| --- | --- | --- | --- | --- | --- | --- |
|  |  | Skin (+)^a^  Other (+)^b^ | Skin (+)  Other (-) | Skin (-)  Other (+) | Skin (-)  Other (-) |  |
| White nails | Restrictive ventilatory dysfunction  (n=40) | 13 (32.5) | 12 (30) | 2 (5) | 13 (32.5) | 0.020 |
|  | Subclinical and clinical hypothyroidism  (n=41) | 19 (46.3) | 6 (14.6) | 6 (14.6) | 10 (24.4) | 0.022 |
|  | Ascites (n=41) | 11 (26.8) | 14 (34.1) | 1 (2.4) | 15 (36.6) | 0.013 |

^a^ Skin (+) refers to appearance of skin changes in the first column. Skin (-) means without skin changes listed in the first column.

^b^ Other (+) refers to appearance of associated symptoms in the second column. Other (-) means without associated symptoms listed in the second column.

Supplementary table 5. Skin responses and relationship with thyroid function improvement at the end of therapy.

| Skin responses | Thyroid function，number of patients (%) | | *P* |
| --- | --- | --- | --- |
|  | Remission^a^ | No remission |  |
| Hyperpigmentation (n=22) | | | |
| Response | 14 (63.6) | 2 (9.1) | **0.025** |
| No response | 2 (9.1) | 4 (18.2) |  |
| Hemangiomas (n=19) | | | |
| Response | 4 (21.1) | 4 (21.1) | 0.111 |
| No response | 10 (52.6) | 1 (5.3) |  |
| Hypertrichosis (n=11) | | | |
| Response | 7 (63.6) | 3 (27.3) | 1.000 |
| No response | 1 (9.1) | 0 (0) |  |

^a^ Remission of thyroid function means recover from clinical (decreased FT4 or TT4 and elevated TSH) or subclinical hypothyroidism (normal FT4 and elevated TSH) to normal thyroid function.

Supplementary table 6. Skin, VEGF, hematological and neurological responses in every patient. ONLS score column shows the change from baseline to the end of therapy.

| No. | Skin responses | | | | | VEGF responses | Hematological responses | ONLS score |
| --- | --- | --- | --- | --- | --- | --- | --- | --- |
|  | Hyperpigmentation | Hypertrichosis | Hemangioma | White nails | Acrocyanosis |  |  |  |
| 1 | R^a^ | — | R | R | — | CR^b^ | CR | 2→1 |
| 2 | R | — | NR^c^ | R | — | CR | N^d^ | 0→0 |
| 3 | R | R | NR | — | R | PR^e^ | CR | 5→4 |
| 4 | NR | R | NR | — | R | PR | CR | 4→1 |
| 5 | R | R | NR | — | — | CR | N | 5→2 |
| 6 | R | R | NR | R | — | PR | N | 6→2 |
| 7 | R | — | — | R | — | CR | N | 3→1 |
| 8 | NR | — | NR | — | — | SD^f^ | N | 3→2 |
| 9 | R | R | — | R | — | CR | N | 3→1 |
| 10 | R | — | R | R | — | CR | N | 9→3 |
| 11 | Died | Died | Died | Died | Died | Died | Died | Died |
| 12 | R | — | NR | R | R | CR | CR | 4→2 |
| 13 | NR | — | — | — | — | SD | N | 6→4 |
| 14 | R | — | NR | R | — | CR | CR | 4→0 |
| 15 | — | — | NR | — | — | CR | CR | 2→2 |
| 16 | NR | — | NR | R | — | SD | N | 1→0 |
| 17 | R | — | — | R | — | CR | CR | 4→2 |
| 18 | R | — | NR | R | R | SD | N | 6→3 |
| 19 | R | — | NR | R | — | PR | N | 3→2 |
| 20 | — | — | NR | — | — | CR | N | 2→1 |
| 21 | R | R | R | — | — | CR | N | 2→1 |
| 22 | R | — | NR | R | — | CR | CR | 6→4 |
| 23 | R | R | NR | R | — | PR | CR | 4→1 |
| 24 | Withdrawal | Withdrawal | Withdrawal | Withdrawal | Withdrawal | Withdrawal | Withdrawal | Withdrawal |
| 25 | R | R | R | R | — | CR | CR | 0→0 |
| 26 | NR | NR | NR | R | — | PR | CR | 5→4 |
| 27 | R | — | NR | — | — | PR | CR | 6→3 |
| 28 | Withdrawal | Withdrawal | Withdrawal | Withdrawal | Withdrawal | Withdrawal | Withdrawal | Withdrawal |
| 29 | R | — | NR | — | — | PR | N | 5→3 |
| 30 | R | — | R | R | — | Normal at baseline | CR | 4→2 |
| 31 | Died | Died | Died | Died | Died | Died | Died | Died |
| 32 | NR | R | R | R | — | CR | CR | 5→3 |
| 33 | NR | — | — | — | — | PR | N | 2→0 |
| 34 | R | R | NR | — | — | PR | N | 3→2 |
| 35 | R | R | — | R | — | CR | N | 9→4 |
| 36 | R | R | R | — | — | CR | CR | 9→6 |
| 37 | NR | R | R | — | — | PR | CR | 3→2 |
| 38 | R | R | R | R | — | PR | N | 9→4 |
| 39 | R | — | R | R | — | PR | CR | 4→2 |
| 40 | Died | Died | Died | Died | Died | Died | Died | Died |
| 41 | R | — | R | — | — | PR | N | 2→1 |

^a^R refers to response. ^b^CR refers to complete responses of VEGF (<600 pg/mL) or M protein (negative immunofixation of the serum and urine). ^c^NR refers to no response. ^d^N refers to not reach complete hematologic response. ^e^PR refers to partial response in VEGF (≥50% reduction but still >600 pg/mL). ^f^SD refers to stable disease in VEGF (not meeting the criteria for CR or PR).
